# Associations Between Schemes of Social Insurance and Self-Rated Health Comparison: Evidence From the Employed Migrants in Urban China

**DOI:** 10.3389/fpubh.2019.00253

**Published:** 2019-09-18

**Authors:** Ming Guan

**Affiliations:** ^1^Family Issues Center, Xuchang University, Xuchang, China; ^2^School of Business, Xuchang University, Xuchang, China

**Keywords:** SRHC, social insurance, health insurance, employed migrants, urban China

## Abstract

**Background:** Little was known about the relationship between social insurance without health insurance and self-rated health comparison (SRHC). The present study aimed to investigate how social insurance schemes improved SRHC among employed migrants in urban China.

**Methods:** The employed migrants aged 18 and above were selected from the 2009 Rural-Urban Migration in China project. Multiple probit regression models were adopted to identify the determinants of participation of social insurance. Multiple logistic regression models were used to analyze the relationship between unemployment insurance, pension insurance, and work injury insurance and SRHC.

**Results:** In the sample, most of the participants were middle-aged, male, and uninsured persons. However, over 80% of them reported better SRHC. Health insurance contributed to the participation of social insurance. The social insurance schemes were associated with financial risk. Regarding the confounding effects of health insurance, the three schemes of social insurance were associated with SRHC.

**Conclusions:** The result indicated that not all three, but two schemes of social insurance, could improve SRHC among the employed migrants.

## Introduction

A large part of the literature has reported the protective effect of health insurance on health status (1–5). Regarding the causal effect of health insurance on health, a review concluded that the observed correlation between insurance and good health might be driven by other factors (6). Two current studies indicated that the effect relied on the health insurance coverage (7, 8). Also, health insurance had some limitations. A study in India revealed that health insurance aggravated the inequities in accessing health care services (9). Similarly, in China, this was also true. Using the 2006 China Agricultural Census, a study found that the New Cooperative Medical System (NCMS) did not affect child morality or maternal mortality (10). The Chinese health insurance policy could not realize full coverage and equal utilization of health insurance among migrant workers in China (11). As for rural-to-urban migrants in China, migration to urban areas limited the effectiveness of

rural health insurance on chronic disease management due to its non-portable nature (12). Thus, social insurance as an alternative method could be used to safeguard the health status.

Currently, little attention has been paid to the impacts of social insurance on health status in academic circles. Similar to the financing system for health insurance in China, the employers are required to make monthly contributions to unemployment insurance (UI), pension insurance (PI), and work injury insurance (WII). Additionally, individuals need to make monthly contributions to UI and PI. Only employers in hazardous industries were required to make monthly WII contributions. WII generally covered all work-related injuries and occupational illnesses. Prior studies reported the relationship between enrollment in the social security system and health status in China (13). Thus, non-medical insurance could be speculated to have a relationship with health change of the insured.

Empirically, a government-run mandatory insurance program could decrease medical care utilization and expenditures. However, it had little impact on self-reported health status (14). Interestingly, it was confirmed that firm-supported insurance schemes protected employees' health. UI mitigated adverse health effects both at individual and country levels during the financial crisis (15). Clinically, UI programs mitigated cardiovascular disease risk (16). From an examination of social insurance coverage and relative concerns, a positive relationship between PI and subjective well-being was observed for the first time in happiness literature on Chinese rural-to-urban migrants (17).

In China, the WII coverage rate was far lower than expected. A current study found the WII coverage was higher than the national average level but much lower than the average international level (18). The awareness of occupational disease and injury insurance was closely associated with WII coverage. Therefore, it was imperative to popularize knowledge on occupational health and safety for migrant workers as well as to elevate the coverage rate of WII.

Recently, very little of the work on the relationship between social insurance schemes and self-rated health comparison (SRHC) has been focused on employed migrant groups in China. Additionally, this study was enlightened by a prior study. For instance, a study suggested that the community-based health insurance in Burkina Faso substantially reduced the likelihood of catastrophic health expenditure (19). This study aimed to fill in the gap, with publicly available survey data covering social insurance schemes among the employed migrants in urban China.

## Methods

### Data Source

Data were from the 2009 Rural-Urban Migration in China (RUMiC) project, which was established to study the patterns and effects of migration in China. It could answer the questions with respect to migration's impact on income mobility, health, and the assimilation of migrant workers into cities.

### Main Variables

The dependent variable was SRHC. SRHC was measured by the question: “Compared to people of the same age as you, how is your current state of health?” Degree of health comparison was measured with an ordinal response scale following five response options: excellent, good, fair, poor, and very poor. In the analysis, this study converted the responses from a five-point scale to a two-point scale by combining excellent and good to “better (= 1),” and fair, poor, and very poor to “worse (= 0).”

There were three schemes of social insurance, including UI, PI, and WII in the questionnaire. UI was reflected by the answer to the question: “Do you have UI?” PI was denoted by the answer to the question: “Do you have PI?” WII was defined by the answer to the question: “Do you have WII?” Their response options to answer these questions were available on a four-point Likert scale: paid by employer, paid by yourself, paid by both employer and yourself, and none. In the analysis, this study converted the responses from a four-point scale to a two-point scale by combining paid by employer, paid by yourself, paid by both employer and yourself to “yes (= 1),” and none to “no (= 0).”

Regarding marital status, married, remarried, and cohabited status were considered as married status (recoded as 1) and divorced, widowed, and never married considered as single status (recoded as 0).

### Main Questions

As reviewed earlier, researchers have correlated social insurance with SRHC. However, it was unclear how schemes of social insurance were associated with SRHC. Thus, the main research questions were below.

Question 1: With regard to financial risk, how was social insurance associated with medical expense?

Question 2: With regard to interactive items of social insurance, how was social insurance associated with SRHC?

Question 3: With regard to health insurance confounders, how was social insurance associated with SRHC?

### Statistical Methods

Here, all the statistical work was conducted by STATA S.E. 14.0 (STATA Corp., Inc., College Station, TX). Chi-square tests were performed to assess whether there were significant gender differences in the sample by demographic factors, social insurance schemes, health insurance schemes, and SRHC.

Predictors of participation of social insurance were computed with socioeconomic factors, life style, health status, and health insurance like commercial medical insurance (CMI), public health insurance (PHI), labor insurance (LI), medical insurance covering family members (MICFM), rural cooperatives medical insurance (RCMI), and comprehensive medical insurance (CHMI) with probit models.

Relationship between social insurance and financial risk were computed with multiple probit models. According to literature (20), health expense were defined as “high” if they exceeded 5% of the sample average monthly income (abbreviated by INCOME, Unit: Chinese Yuan), and as “catastrophic” if they exceeded 10% of the average monthly income.

The positive health effects of social insurance possibly resulted from participation in health insurance. Thus, the confounding effects of health insurance and interactive effects of social insurance were considered.

Considering confounding factors in a stepwise fashion, the association between social insurance and SRHC was assessed using logistic regression models. The *chest* command in Stata was used to examine confounding effects for total, males, and females separately. The socioeconomic factors, lifestyle, health status, and health insurance were considered as potential confounding variables. When measuring mediating effects, change-in-estimate criterion with a 0.09% cutoff was adopted (21, 22).

Analyzing how the SRHC in the employed migrants was affected by the schemes of social insurance participation with logistic regressions, partial effects in probit models with a triple dummy-variable interaction term could be derived with Stata *inteff3* command (23). Simultaneously, socioeconomic factors, lifestyle, health status, and health insurance were considered as control variables. Here, interactions between the schemes of insurance were adopted to reflect social insurance enrollment portfolio. For convenience, the symbol “×” denoted interactive computation. Thus, interaction terms like UI × PI, UI × WII, WII × PI, and WII × UI × PI could be covariates.

## Results

### Subjects

The respondents were selected from the 2009 RUMiC project on the basis of migrants in urban China. As seen in Table 1, the average age of the sample was 32.53 (*SD* = 10.237, min = 18, max = 72, *n* = 7,176). Overall, 4,218 (58.63%) were male, 2,976 (41.37%) were female. Considering insurance, 884 (12.54%, *n* = 7,051) were covered by UI; 1,484 (20.95%, *n* = 7,084) were covered by PI; and 1,199 (17.03%, *n* = 7,039) were covered by WII. Likewise, 5,817 (80.86%, *n* = 7,194) reported better SRHC. There were significant gender differences in the case of age, marital status, body mass index (BMI) categories, UI, PI, WII, and LI.

**Table 1:** Sample characteristics by gender.

**Variables**	**Male**		**Female**		**Chi2**	***P*-value**
	***N***	**%**	***N***	**%**		
Age (*n* = 7,176)					34.0493	0.000^***^
18–29	1,873	26.10	1,381	19.24		
30–39	1,241	17.29	862	12.01		
40–49	773	10.77	589	8.21		
50–	326	4.54	131	1.83		
Marital status (*n* = 7,192)					14.0598	0.000^***^
Single	1,482	20.61	919	12.78		
Married	2,736	38.04	2,055	28.57		
BMI Categories (*n* = 7,156)					214.0485	0.000^***^
Underweight	247	3.45	454	6.34		
Normal	3,129	43.73	2,139	29.89		
Overweight	763	10.66	337	4.71		
Obesity	58	0.81	29	0.41		
Physical disabilities (*n* = 7,194)					0.6655	0.415
Yes	160	2.22	102	1.42		
No	4,058	56.41	2,874	39.95		
High expense (*n* = 1,098)					0.2837	0.594
Yes	586	53.37	433	39.44		
No	43	3.92	36	3.28		
Catastrophic expense (*n* = 1,098)					0.2837	0.594
Yes	586	53.37	433	39.44		
No	43	3.92	36	3.28		
UI (*n* = 7,051)					11.7576	0.001^***^
No	3,567	50.59	2,600	36.87		
Yes	565	8.01	319	4.52		
PI (*n* = 7,084)					25.3775	0.000^***^
No	3,202	45.20	2,398	33.85		
Yes	956	13.50	528	7.45		
WII (*n* = 7,039)					64.4566	0.000^***^
No	3,306	46.97	2,534	36.00		
Yes	829	11.78	370	5.26		
CMI (*n* = 7,193)					0.3679	0.544
No	4,042	56.19	2,861	39.77		
Yes	175	2.43	115	1.60		
PHI (*n* = 7,193)					2.3122	0.128
No	4,128	57.39	2,928	40.71		
Yes	89	1.24	48	0.67		
LI (*n* = 7,193)					12.2432	0.000^***^
No	3,865	53.73	2,793	38.83		
Yes	352	4.89	183	2.54		
MICFM (*n* = 7,193)					0.8081	0.369
No	4,192	58.28	2,963	41.19		
Yes	25	0.35	13	0.18		
RCMI (*n* = 7,193)					0.2528	0.615
No	1,783	24.79	1,276	17.74		
Yes	2,434	33.84	1,700	23.63		
CHMI (*n* = 7,193)					0.7362	0.391
No	4,170	57.97	2,949	41.00		
Yes	47	0.65	27	0.38		
SRHC (*n* = 7,194)					1.8522	0.174
No	785	10.91	592	8.23		
Yes	3,433	47.72	2,384	33.14		

*** *p <0.01*.

*SRHC, self-rated health comparison; UI, unemployment insurance; PI, pension insurance; WII, work injury insurance; CMI, commercial medical insurance; PHI, public health insurance; LI, labor insurance; MICFM, medical insurance covering family members; RCMI, rural cooperatives medical insurance; CHMI, comprehensive medical insurance*.

### Predicting Participation of Social Insurance

In Table 2, gender, CMI, PHI, LI, and CHMI were significantly associated with social insurance. BMI was significantly associated with social insurance. Marital status was significantly associated with PI. Age and physical disabilities were significantly associated with WII and UI. MICFM was significantly associated with WII and PI. This suggested that health insurance schemes would promote the participation possibilities of the schemes of social insurance of the employed migrants. But, age, BMI, and physical disabilities decreased participation possibilities for the social insurance schemes. Males and females differed in the participation in the social insurance schemes. With respect to health insurance, CMI, PHI, LI, and CHMI increased the participation of the schemes of social insurance. The employed migrants with MICFM were more likely to participate in WII and PI, while the employed migrants with physical disabilities were less likely to participate in WII and UI. Thus, the health insurance schemes could predict the possibilities of participation of the schemes of social insurance.

**Table 2:** Predictors of participation of social insurance.

	**WII**	**PI**	**UI**
Age	−0.01^***^ (0.00)	−0.00 (0.00)	−0.01^***^ (0.00)
Gender	0.32^***^ (0.04)	0.14^***^ (0.04)	0.12^***^ (0.04)
Marital status	0.01 (0.05)	0.11^**^ (0.05)	0.05 (0.06)
BMI	−0.05^***^ (0.00)	−0.05^***^ (0.00)	−0.05^***^ (0.00)
Physical disabilities	−0.38^***^ (0.13)	−0.08 (0.10)	−0.34^**^ (0.14)
SRHC	0.02 (0.05)	−0.05 (0.05)	−0.05 (0.05)
INCOME	−0.00 (0.00)	−0.00 (0.00)	−0.00 (0.00)
CMI	1.04^***^ (0.09)	1.46^***^ (0.10)	1.02^***^ (0.09)
PHI	1.29^***^ (0.13)	1.48^***^ (0.14)	1.27^***^ (0.12)
LI	1.77^***^ (0.07)	1.96^***^ (0.08)	1.46^***^ (0.07)
MICFM	0.47^*^ (0.27)	0.60^**^ (0.25)	0.24 (0.27)
RCMI	0.06 (0.04)	−0.03 (0.04)	−0.02 (0.04)
CHMI	1.95^***^ (0.18)	1.85^***^ (0.24)	1.72^***^ (0.19)
Observations	6,859	6,900	6,868

* *p <0.1*,

** *p <0.05*,

*** *p <0.01*.

*SRHC, self-rated health comparison; UI, unemployment insurance; PI, pension insurance; WII, work injury insurance; CMI, commercial medical insurance; PHI, public health insurance; LI, labor insurance; MICFM, medical insurance covering family members; RCMI, rural cooperatives medical insurance; CHMI, comprehensive medical insurance*.

Different from the early studies that health status might contribute to the participation of social health insurance (24) and the enrollment choice of the community-based health insurance scheme (25), this study found SRHC was not the predictor of participation of social insurance.

### Associations Between Social Insurance and Financial Risk

In Table 3, UI raised the probabilities of “high” and “catastrophic” medical expenses by 0.66% in the female sample. PI reduced the probabilities of “high” and “catastrophic” medical expenses by 0.94% in the female sample. WII raised the probabilities of high and catastrophic medical expense by 0.39% and 0.61% in female and male sample, respectively.

**Table 3:** Associations between schemes of social insurance and high expense and catastrophic expense.

	**High expense**			**Catastrophic expense**		
	**Total**	**Female**	**Male**	**Total**	**Female**	**Male**
Age	−0.01^*^ (0.01)	−0.01 (0.01)	−0.01 (0.01)	−0.01^*^ (0.01)	−0.01^***^ (0.01)	−0.01 (0.01)
Gender	0.02 (0.13)	−0.84^***^ (0.28)	−0.62^***^ (0.21)	0.02 (0.13)	−0.84 (0.28)	−0.62^***^ (0.21)
Marital status	−0.65^***^ (0.17)	0.12^***^ (0.02)	0.09^***^ (0.01)	−0.65^***^ (0.17)	0.12^***^ (0.02)	0.09^***^ (0.01)
BMI	0.10^***^ (0.01)	−0.87^**^ (0.44)	0.30 (0.54)	0.10^***^ (0.01)	−0.87^**^ (0.44)	0.30 (0.54)
Physical disabilities	−0.28 (0.32)	0.49 (0.50)	0.14 (0.41)	−0.28 (0.32)	0.49 (0.50)	0.14 (0.41)
UI	0.17 (0.32)	0.66^**^ (0.32)	0.03 (0.29)	0.17 (0.32)	0.66^**^ (0.32)	0.03 (0.29)
PI	0.30 (0.23)	−0.94^*^ (0.50)	0.29 (0.33)	0.30 (0.23)	−0.94^*^ (0.50)	0.29 (0.33)
WII	−0.12 (0.27)	0.39^**^ (0.20)	0.61^***^ (0.17)	−0.12 (0.27)	0.39^**^ (0.20)	0.61^***^ (0.17)
SRHC	0.52^***^ (0.13)	−0.00 (0.00)	−0.00 (0.00)	0.52^***^ (0.13)	−0.00 (0.00)	−0.00 (0.00)
INCOME	−0.00 (0.00)	−0.52 (0.37)	−0.88^**^ (0.35)	−0.00 (0.00)	−0.52 (0.37)	−0.88^**^ (0.35)
CMI	−0.76^***^ (0.25)			−0.76^***^ (0.25)		
PHI		−0.40 (0.34)	−0.25 (0.33)		−0.40 (0.34)	−0.25 (0.33)
LI	−0.33 (0.24)		−1.24^**^ (0.48)	−0.33 (0.24)		−1.24^**^ (0.48)
MICFM	−0.92^**^ (0.43)	−0.10 (0.21)	0.13 (0.19)	−0.92^**^ (0.43)	−0.10 (0.21)	0.13 (0.19)
RCMI	0.06 (0.14)			0.06 (0.14)		

* *p <0.1*,

** *p <0.05*,

*** *p <0.01*.

*SRHC, self-rated health comparison; UI, unemployment insurance; PI, pension insurance; WII, work injury insurance; CMI, commercial medical insurance; PHI, public health insurance; LI, labor insurance; MICFM, medical insurance covering family members; RCMI, rural cooperatives medical insurance; CHMI, comprehensive medical insurance*.

In the total sample, age was negatively associated with an increase in high expense in the total sample and catastrophic expense in the total and female samples. Gender was negatively associated with an increase in high expense in the female and male samples and catastrophic expense in the male sample. Marital status was negatively associated with an increase in high and catastrophic expense in the total sample and positively associated with an increase in high and catastrophic expense in the female and male samples. However, BMI was positively associated with an increase in high and catastrophic expense in the total sample and negatively associated with an increase in high and catastrophic expense in the female sample. There was no significant effect of health insurance on the probabilities of incurring high and catastrophic health expenses in the total, female, or male samples.

Among the schemes of health insurance, there were negatively significant effects of CMI and MICFM on the probabilities of incurring high and catastrophic health expenses in the total sample. Similarly, LI was negatively associated with an increase in high and catastrophic expense in the male sample. There were no significant effects of PHI or RCMI on the probabilities of incurring high and catastrophic health expenses in the total, female, or male samples.

### Association Between Social Insurance and SRHC Regarding Health Insurance

Table 4 showed that the change-in-estimate of BMI, MICFM, and CMI were lower than the 0.09% cutoff criterion, which indicated they were covariates. However, the change-in-estimate of RCMI, INCOME, physical disabilities, CHMI, gender, marital status, LI, PHI, PI, and WII were higher than the 0.12% cutoff criterion, which indicated they were potential confounding variables.

**Table 4:** Change-in-estimate for UI with possible confounding factors.

	**OR**	**95% CI**	**Change, %**
Adj.All	0.8698	0.6458–1.1714	
–MICFM	0.8697	0.6458–1.1711	0.0161
–CMI	0.8690	0.6452–1.1704	0.0740
–BMI	0.8698	0.6459–1.1713	0.0859
–RCMI	0.8708	0.6465–1.1730	0.1225
–Average monthly income	0.8696	0.6455–1.1715	0.1412
–Physical disabilities	0.8674	0.6440–1.1683	0.2530
–CHMI	0.8698	0.6456–1.1718	0.2806
–Gender	0.8669	0.6435–1.1679	0.3318
–Marital status	0.8693	0.6465–1.1689	0.2750
–LI	0.8616	0.6406–1.1588	0.8881
–PHI	0.8687	0.6468–1.1666	0.8191
–PI	0.8936	0.6821–1.1707	2.8722
–Age	0.8944	0.6866–1.1651	0.0898
–WII	1.2270	1.0106–1.4899	37.1900

Table 5 showed that the change-in-estimate of gender, BMI, and MICFM were lower than the 0.09% cutoff criterion, which indicated they were covariates. However, the change-in-estimate of RCMI, CHMI, physical disabilities, INCOME, CMI, marital status, PHI, UI, LI, age, and WII were higher than the 0.11% cutoff criterion, which indicated they were potential confounding variables.

**Table 5:** Change-in-estimate for PI with possible confounding factors.

	**OR**	**95% CI**	**Change, %**
Adj.All	1.0859	0.8450–1.3955	
–Gender	1.0857	0.8448–1.3952	0.0207
–MICFM	1.0860	0.8454–1.3951	0.0300
–BMI	1.0864	0.8458–1.3954	0.0328
–RCMI	1.0875	0.8465–1.3971	0.1060
–CHMI	1.0903	0.8485–1.4010	0.2586
–Physical disabilities	1.0930	0.8508–1.4042	0.2479
–Average monthly income	1.0977	0.8550–1.4093	0.4253
–CMI	1.0916	0.8545–1.3945	0.5555
–Marital status	1.0813	0.8473–1.3799	0.9407
–PHI	1.1100	0.8723–1.4125	2.6531
–UI	1.0735	0.8606–1.3390	3.2900
–LI	1.0244	0.8282–1.2670	4.5737
–Age	0.9660	0.7809–1.1949	5.7026
–WII	1.2016	1.0282–1.4042	24.3918

Table 6 showed that the change-in-estimate of INCOME, MICFM, and CMI were lower than the 0.09% cutoff criterion, which indicated they were covariates. However, the change-in-estimate of BMI, CHMI, RCMI, physical disabilities, marital status, PHI, gender, LI, PI, UI, and age were higher than the 0.12% cutoff criterion, which indicated they were potential confounding variables.

**Table 6:** Change-in-estimate for WII with possible confounding factors.

	**OR**	**95% CI**	**Change, %**
Adj.All	1.3645	1.0363–1.7965	
–MICFM	1.3646	1.0366–1.7965	0.0105
–Average monthly income	1.3645	1.0361–1.7970	0.0108
–CMI	1.3636	1.0351–1.7965	0.0620
–BMI	1.3609	1.0331–1.7926	0.2024
–CHMI	1.3637	1.0367–1.7938	0.2054
–RCMI	1.3588	1.0330–1.7873	0.3583
–Physical disabilities	1.3493	1.0264–1.7737	0.6996
–Marital status	1.3591	1.0364–1.7824	0.7315
–PHI	1.3703	1.0466–1.7942	0.8243
–Gender	1.3874	1.0609–1.8142	1.2427
–LI	1.3391	1.0259–1.7479	3.4805
–PI	1.3776	1.0799–1.7574	2.8756
–UI	1.2817	1.0739–1.5296	6.9642
–Age	1.3840	1.1614–1.6493	7.9840

In Table 7, controlling for RCMI, INCOME, physical disabilities, CHMI, gender, marital status, LI, PHI, PI, and WII, UI was significantly associated with SRHC (OR = 1.24, 95%CI: 1.02–1.51). Controlling for RCMI, CHMI, physical disabilities, INCOME, CMI, marital status, PHI, UI, LI, age, and WII, PI was significantly associated with SRHC (OR = 1.24, 95%CI: 1.07–1.45). Controlling for BMI, CHMI, RCMI, physical disabilities, marital status, PHI, gender, LI, PI, UI, and age, WII was significantly associated with SRHC (OR = 1.72, 95%CI: 1.19–2.48). In model 1, the aged migrants tended to be with worse health status. Moreover, the health effect appeared to be stronger for the employed migrants with higher BMI and those with more expenses than their counterparts.

**Table 7:** SRHC for social insurance without confounders, OR (95%CI).

	**Model 1**	**Model 2**	**Model 3**
UI	Ref. = No		
Yes	1.24^**^ (1.02–1.51)		
PI		Ref. = No	
Yes		1.24^***^ (1.07–1.45)	
WII			Ref. = No
Yes			1.72^***^ (1.19–2.48)
Age	0.97^***^ (0.97–0.98)		
Gender		Ref. = Female	
Male		1.09 (0.97–1.23)	
BMI	1.11^***^ (1.10–1.12)	1.06^***^ (1.06–1.07)	
High expense			Ref. = No
Yes			1.45^***^ (1.26–1.66)
MICFM	Ref. = No	Ref. = No	Ref. = No
Yes	1.26 (0.51–3.12)	1.26 (0.52–3.08)	1.57 (0.46–5.36)
CMI	Ref. = No		Ref. = No
Yes	1.06 (0.77–1.48)		1.39 (0.78–2.47)

** *p <0.05*,

*** *p <0.01*.

*SRHC, self-rated health comparison; UI, unemployment insurance; PI, pension insurance; WII, work injury insurance; CMI, commercial medical insurance; MICFM, medical insurance covering family members*.

### Association Between Interactions Between Schemes of Social Insurance and SRHC

In Table 8, the effect of the variable WII showed that the probability of WII to have better health status was about 4.2% points higher than that not covered by WII.

**Table 8:** Association between social insurance portfolio and SRHC.

	**Total**	**Female**	**Male**
UI	−0.030 (0.052)	0.023 (0.073)	−0.093 (0.079)
PI	0.008 (0.019)	0.035 (0.029)	0.000 (0.025)
WII	0.042^*^ (0.024)	−0.013 (0.053)	0.071^**^ (0.027)
UI × PI	0.082 (0.078)	−0.007 (0.107)	0.190 (0.118)
UI × WII	−0.162^*^ (0.096)	−0.417^**^ (0.181)	−0.016 (0.127)
PI × WII	0.023 (0.040)	0.182^**^ (0.080)	−0.042 (0.048)
UI × PI × WII	0.113 (0.136)	0.334 (0.227)	−0.062 (0.185)

* *p <0.1*,

** *p <0.05*.

*SRHC, self-rated health comparison; UI, unemployment insurance; PI, pension insurance; WII, work injury insurance*.

Among the female sample, the interaction effect −0.182 of PI and WII (PI × WII) meant that (1) the PI difference was 18.2% points higher for average individuals with WII compared with similar individuals without WII, or that (2) the negative effect of WII participation was 18.2% points higher for individuals with PI than it was for individuals without PI.

Among the total sample, the effect for UI × WII showed that (1) for the employed migrants with WII, the UI difference was 16.2% points lower than for the employed migrants without WII, or (2) for the employed migrants with UI, the negative effect of WII on SRHC was 16.2% points lower than it was for the employed migrants without UI.

Among the female sample, the effect for UI × WII showed that (1) for the employed migrants with WII, the UI difference was 41.7% points smaller than for the employed migrants without WII, or (2) for the employed migrants with UI, the negative effect of WII on SRHC was 41.7% points lower than it was for the employed migrants without UI.

The insignificant effect of UI × PI × WII implied that (1) the effect of UI on PI did not seem to depend on WII, (2) the effect of UI on WII did not seem to depend on PI, or (3) the effect of PI on WII did not seem to depend on UI.

## Discussion

To my knowledge, this was the first study to report the relationship between social insurance schemes and SRHC among employed migrants in urban China. Considering participation of the social insurance schemes, besides socio-demographic and lifestyle variables, health insurance schemes contributed significantly. Regarding financial risk, UI and WII could increase medical expenditure while PI could decrease the medical expense. Controlling for the schemes of health insurance, social insurance schemes were associated with SRHC. In the terms of interactive effects, two schemes of social insurance interaction terms had marginal effects at the means of probit estimation.

The results in this study were in line with an early study. The socioeconomic factors influenced the participation of rural migrant workers in social insurance systems. However, the relative importance of these factors in participation in social insurance was differentiated by gender (26). Also, the AORs of the UI × WII were less than the AORs of PI × WII. This suggested that PI would shorten the health contribution of social insurance schemes.

Although two studies confirmed health insurance could reduce medical expenditures (27–31), this study was not in line with the prior study regarding the role of health insurance in China. For example, an analysis concluded NCMS had failed to prevent catastrophic health expenditure and medical impoverishment (32) and could not lead to better self-assessed general health status in rural China (33). Using data from the 2015 China Migrants Dynamic Survey, it was observed that participation in the health insurance system significantly improved floating seniors' self-rated health (34). This could be partly explained by an early study that a small percentage of households with health insurance coverage were unable to self-finance out-of-pocket medical cost, particularly for inpatient treatments and/or chronic diseases in China (35).

The interaction analysis possibly implied that the social insurance system played an important role in participants' health improvement. This was congruent with several studies that attached importance to social insurance officers (36, 37). Individual health improvement of the insured workers might also be negatively affected by information exchange with social insurance physicians and occupational physicians (38), insurance underutilization (39), poor personal contact with social insurance officers and health care professionals (40), and low use of evidence-based medicine and clinical practice guidelines in insurance medicine (41). Another study showed that the interaction with the social insurance agency and health care could be considered as an important part of the rehabilitation process (42). A current study that used data from the 2014 National Internal Migrant Dynamic Monitoring Survey confirmed the positive financial protection effect of social health insurance among rural-to-urban internal migrants in China (43).

Here, there was little likelihood that the majority of migrant workers who moved to cities were able to access the social insurance benefits traditionally available to those with urban household registration. This result was in line with the early result that the migrants, the poor and the vulnerable remained in the edge of the public, private and multiple insurance coverage system (44). It could be explained in the case of socioeconomic factors in Jiangsu Survey Data (45), exclusion from urban residents (46), employers' attitudes toward social insurance compliance (47), and large disparities between firm ownership sectors in social insurance participation and generosity (48). Also, migrants' limited access to health insurance owed more to their reluctance than to system exclusions (49). However, the results in this study provided new insights.

This study presented the idea of interaction effect of social insurance among the employed migrants. This judgment was congruent with another early study. In fact, possibly due to the lack of systematic financing schemes, medical insurance programs were not effective in alleviating the financial burden of healthcare and promoting formal medical utilization among migrant workers (50). Another example showed that the disharmonized social insurance systems caused insured clients to wait unnecessarily in Sweden (51). Thus, the existence of the interaction effect and order of enrollment in insurance schemes could help the employed save economic cost.

## Limitations and Strengths

This study had two main limitations. First, dynamic benefits of combinations of insurance schemes could not be reflected in a cross-sectional data. Second, subjective assessments among the employed migrants necessarily need support from objective criteria.

The strength of this study was that advanced statistical methods were adopted through reducing confounding effects of health insurance to explore the associations between social insurance and SRHC.

## Future Research

This study could not reveal why socio-demographic factors statistically reduced the contribution of insurance schemes to SRHC among employed migrants. Further exploration should focus on the moderating and mediating effects of socioeconomic factors on the change of health status among employed migrants.

## Conclusion

In conclusion, SRHC of employed migrants in urban China was found to be affected by social insurance. This study also confirmed that the effects of social insurance on SRHC were mediated by the health insurance of employed migrants. This study still highlighted the interaction effect in social insurance schemes for the employed migrants. These findings suggested that individuals might choose to optimize insurance schemes in order to obtain optimal health status. This study also provided implications on reforming China's social insurance system.

## Data Availability

The dataset 2009 RUMiC project for this study can be found in the Institute for the Study of Labor [http://idsc.iza.org/rumic].

## Author Contributions

MG conceived the study, performed statistical analyses, drafted the manuscript, read, and approved the final manuscript.

### Conflict of Interest Statement

The author declares that the research was conducted in the absence of any commercial or financial relationships that could be construed as a potential conflict of interest.

## Abbreviations

SRHC: self-rated health comparison
RUMiC: Rural-Urban Migration in China
AOR: adjusted odd ratio
CI: confidence interval
UI: unemployment insurance
PI: pension insurance
WII: work injury insurance
CMI: commercial medical insurance
PHI: public health insurance
LI: labor insurance
MICFM: medical insurance covering family members
RCMI: rural cooperatives medical insurance
CHMI: comprehensive medical insurance.
